# Editorial Department

**Published:** 1914-03

**Authors:** 


### EDITORIAL DEPARTMENT.

**DR. F. E. DANIEL, Editor** **DR. R. H. L. BIBB, Associate Editor**

DEPARTMENTS { EUGENICS: Drs. M. Duggan and T. Y. Hull.

WOMAN'S DEPARTMENT: Mrs. F. E. Daniel.

OWING TO THE ILLNESS of Dr. Daniel we are without his usual quota of editorial this month. We have endeavored, however, to give you "something just as good."

DEATH OF DR. MCFARLAND.—Dr. T. J. McFarland, an eminent surgeon and physician and a distinguished Confederate surgeon, died at his home at Port Lavaca, Texas, February 21 (ult.) in his 78th year. News of his death and biographical data received too late for a more extended notice. A suitable tribute will appear in our next issue.

DR. J. H. EVANS, President Texas State Board of Medical Examiners, died suddenly at his home in Palestine, February 24th (ult.) His death is much regretted, as he was one of the most valuable workers in the Association, universally liked and esteemed. I was unable to get data and photo for a biographical sketch for this issue, as application by letter to his family remains till now unanswered.

